# Microbial wars: Competition in ecological niches and within the microbiome

**DOI:** 10.15698/mic2018.05.628

**Published:** 2018-05-07

**Authors:** Maria A. Bauer, Katharina Kainz, Didac Carmona-Gutierrez, Frank Madeo

**Affiliations:** 1 Institute of Molecular Biosciences, University of Graz, Graz, Austria.; 2 BioTechMed Graz, Graz, Austria.

**Keywords:** passive competition, active competition, microbiome, selection, niche, probiotics, antibiotics, ecological stability, Candida, Escherichia coli

## Abstract

Many microbial communities live in highly competitive surroundings, in which the fight for resources determines their survival and genetic persistence. Humans live in a close relationship with microbial communities, which includes the health- and disease-determining interactions with our microbiome. Accordingly, the understanding of microbial competitive activities are essential at physiological and pathophysiological levels. Here we provide a brief overview on microbial competition and discuss some of its roles and consequences that directly affect humans.

The earth is inhabited by a vast quantity of diverse microorganisms. The relationship between them is determined by the fundamental drive of each species and strain to promote its own survival. For instance, some strains may live in tightly associated up to symbiotic relationships, thus heavily relying on their allies. Conversely, others may engage in ferocious competition, resulting in a relentless war to win over finite resources such as nutrients, light or territory.

According to Ghoul and Mitri, a strain is competitive if it shows phenotypes that cause a fitness decrease in a competitor strain 1. Characteristically, such phenotypes (e.g., secretion of digestive enzymes, production of antibiotics or inhibition of quorum sensing) have mostly evolved because of biotic competition rather than environmental pressure. Competing strains may be distantly related species or, in contrast, only differ by a single mutation: to be competitors, they must but overlap in resource use. If that is the case, competition may be either passive or active. During passive competition, one strain harms another one through resource consumption, whereas during active competition, individual cells of two contending strains damage one another through direct and active interference 1.

In the frame of passive competition, a strain may restrict the competitor’s access to nutrients, e.g*.* via secretion of digestive enzymes 2,3 or siderophores 4,5. It may also enhance its own nutrient utilization by altering its metabolic regulation 6. Strains may also gain advantage through reduced expression of costly genes by exploiting the expression products of other strains, often referred to as "cheating" 2,4,7. Finally, microorganisms can compete passively by gaining enhanced access to a given space. This may occur through production of molecules that impact space structure and/or microbial motility, such as surfactans, rhamnolipids, adhesion and anti-adhesion molecules or extracellular polysaccharids 8,9,10,11,12.

In settings of active competition (or "interference competition"), rival cells damage each other actively or through chemical warfare with the final goal to eliminate the competitor 1. One strategy to achieve this goal includes using the contact-dependent type IV secretion system, whereby cells inject toxins or other molecules into neighbouring adversaries to promote cell lysis 13,14,15. In fact, the production of antimicrobials ranging from strain-specific bacteriocins to more broad-spectrum antibiotics and peptides is the classical example of interference competiton. A more subtle (but also effective) strategy used for active competition is the active disruption of signalling molecules through quorum quenching 16,17.

The evolutionary emergence and preservation of numerous competition strategies reflect their central role in the microbial ecosystem. But is competition also common? Do microorganisms fight each other exceptionally or routinely? Interestingly, quantification attempts regarding the competitive properties of microbes show, for example, that 25% of gram-negative bacteria possess genes coding for a Type VI secretion system 18. Also, 5-10% of the genomes of nearly all actinomycetes code for secondary metabolites 19, including antibiotics or other possibly damaging molecules. However, the function of these metabolites and the percentage that is actually aggressive still needs to be revealed. Moreover, similar analyses in other microbial groups are still pending. In addition, the occurrence of competition depends on the prevailing environmental conditions. Criteria that promote competition include (i) a high overlap between coexisting strains in their metabolic and/or spatial niche accompanied by the requirement of similar resources, (ii) a relatively high cell density rate relative to the available resources leading to their limitation and (iii) an adequate intermixture of populations, which increases the possibility of interaction, shared nutrients and joint secretions 1,20,21,22.

In general, it is difficult to address the complexity of a microbial community, which covers many influencing components, ranging from environmental to genetic factors. Accordingly, competitive aspects of such communities are duly intricate. In an attempt to assess the extent of exploitative competition, simulations of competitive activity using theoretical metabolic models of different bacteria have been run based on available sequence data 23,24. Despite the limitations of such simulated interactions (*e.g.* no consideration of the natural environment or actual expression of identified genes), they do provide relevant insight into competitive dynamics. One of the early studies using this approach pre-estimates excessive competition between many bacterial species but few cooperative interactions 23. Additionally, many other studies support the prevalence of competition, which has been recently reviewed 1.

An example for the complexity of microbial communities is the human microbiome. Its different microbial societies need to function steadily in order to support the host’s life and health for the final sake of the individual strains’ survival. Hence, ecological stability in the different microbiota in and on our bodies is essential. But how does competition influence the stability of a microbial community? Generally, it increases ecological stability as well as it leads to a local decrease of microbial diversity 25, "local" meaning the range in which microorganisms influence each other’s fitness. Of note, bacteriophages can impact microbial competition and promote diversity by driving horizontal gene transfer and giving rarer species an advantage 26. Horizontal gene transfer occurs rarely but can have major effects by moving single functions (e.g. antibiotic resistance) horizontally through different strains and species 27,28.

There are three established possible long-term consequences of microbial competiton that are ecologically stable 1:

a) *A winner is declared.* One competitive strain dominates the community whereas the "weaker" strain goes extinct 29,30.

b) *A metabolic niche is established.* The competitors are able to coexist because they specialize on different resource types and therefore occupy different metabolic niches. For instance, when distinct bacterial isolates of tree-holes (aquatic ecosystems created for example by a deformed trunk) are brought into co-culture, they initially tend to compete with one another. Eventually, however, they diverge in their use of resources (e.g*.* using each other’s waste products) as they coevolve 31,32. This leads to a niche differentiation where finally competition leads to symbiotic relationships or productive communities 32.

c) *Territorial niches are assigned.* The competitors separate into different territorial niches or patches. This outcome may happen in solid or semi-solid structures such as the soil, mucus or an agar surfaces. This phenomenon has been extensively investigated for microbial colonies, which start as well mixed competitive populations and finally form clonal patches onto the agar surface 33,34.

As mentioned above, competition - with some exceptions - reduces diversity but increases local ecological stability. However, the resulting long-term outcomes in each competitive scenario likely depends on the selection pressures of the surrounding environment. Of note, selection may lead to a microbial arms race, competitive exclusion as well as synergistic division of labour, all in the same environment but in different areas 35. Exactly how diversity, stability and the prevalence of competition and cooperation is influenced needs to be further addressed in future research 1.

Interestingly, in this issue of *Microbial Cell*, Cabral *et al.* address the intestinal microbiome’s ecological stability in a broader sense 36. The intestinal microbiome is a complex community of bacteria, viruses, fungi and some parasitic eukaryotes 37. This complex community and its interactions have co-evolved, maintaining relative homeostasis, which is beneficial for the host as well as for each strain within its niche 37. Therefore, it is of utmost importance for the host but also for every established part of the microbiome that the communal equilibrium remains stable 38. As mentioned, competition promotes ecological stability, which also applies for the intestinal microbiome 25. Interestingly, Cabral *et al.* found *Escherichia coli* to outcompete and kill the opportunistic fungal pathogen *Candida albicans* *in vitro* by secreting a soluble fungicidal factor 36. This factor is inhibited by magnesium, and future work will address the connection between intestinal changes in magnesium levels and microbial homeostasis. Importantly, the secretion of this fungicidal factor could be one of the strategies that have evolved in the intestinal microbiome to prevent Candida overgrowth 39. *C. albicans* is found in the gastrointestinal flora of most healthy humans and was long thought to exist in our gut merely as a commensal resident or as an opportunistic pathogen. Indeed, *C. albicans* is responsible for more than 400.000 life-threatening infections per year, worldwide 40. However, additional roles of *C. albicans* within the gut are becoming evident that emerge from its interaction with bacterial communities. On the one hand, it is implicated in the promotion of bacterial host colonization and virulence upon bacterial co-infection 41. On the other hand, recent findings suggest that *C. albicans* might have beneficial effects for the host upon intestinal infections with bacterial pathogens through an increase of a protective immune response in the host 42. Altogether, intestinal Candida-bacteria (competitive) interactions certainly contribute to human health and disease.

The identification of a novel fungicidal factor by Cabral *et al.* exemplifies the potential of such investigations to uncover possible new treatment strategies or drugs against dangerous infections. In fact, the study of microbial competition (within our bodies and in the environment) engages many areas of biology and medicine. For instance, competition between co-evolving bacterial strains may lead to antibiotic resistance 43, and the development of biofilms, promoted by bacterial competition, also hampers the effective treatment of bacterial infections 44. Given that antibiotic resistance has developed into a global medical threat, mainly due to an irresponsible use of antibiotics, a deeper understanding of how microbial competition contributes to this problem is needed 45. Moreover, gut dysbiosis may be treated by the (personalized) administration of probiotics 46, microorganisms that provide health benefits when consumed 47. For the successful design of new, functional probiotics, more knowledge about how bacteria compete is warranted. If a probiotic strain is not competitive in all ecosystems it will only occasionally implement its desired function 48. Hence, understanding competition ameliorates the chances to cure dysbiosis. For example, it has been proposed that probiotic action can be enhanced by making the gut temporarily susceptible to invasion 48. Other examples for the importance to study microbial competition include industrial applications, e.g. in animal husbandry 49 and evolutionary aspects. In fact, studying microbial competition in the context of group level processes helps to gain insight into the competition-driven evolutionary transition to multicellularity 50.

Charles Darwin noted that "If it could be proved that any part of the structure of any one species had been formed for the exclusive good of another species, it would annihilate my theory, for such could not have been produced through natural selection." 51. Accordingly, microbial wars are an integral part of interspecies equilibrium that affect us both through our environment and within our own microbiome. Further understanding of these competitive interactions will thus not only provide deep insight into different aspects of biology but also shape new advancements in therapeutic approaches.
